# Rethinking psychosocial rehabilitation in mental health

**DOI:** 10.1371/journal.pmed.1005285

**Published:** 2026-11-10

**Authors:** Eleonora Prina, Laura Asher, Stefano Barlati, Sudipto Chatterjee, Ioana Alina Cristea, Sandra Ferreira, Charlotte Hanlon, Karli Montague-Cardoso, Parth Sharma, Marianna Purgato, Corrado Barbui

**Affiliations:** 1 WHO Collaborating Centre for Research and Training in Mental Health and Service Evaluation, Department of Neurosciences, Biomedicine and Movement Sciences, Section of Psychiatry, University of Verona, Verona, Italy; 2 Nottingham Centre for Public Health and Epidemiology, School of Medicine, University of Nottingham, Nottingham, United Kingdom; 3 Institute of Mental Health, University of Nottingham, Nottingham, United Kingdom; 4 Department of Clinical and Experimental Sciences, University of Brescia, Brescia, Italy; 5 Mental Health Matters, Bengaluru, India; 6 Department of General Psychology, University of Padua, Padua, Italy; 7 aves Mental Health, Paarl, Western Cape, South Africa; 8 Division of Psychiatry, Institute for Neuroscience and Cardiovascular Research, University of Edinburgh, Edinburgh, Scotland, United Kingdom; 9 Department of Psychiatry and WHO Collaborating Centre for Mental Health Research and Capacity Building, School of Medicine, College of Health Sciences, Addis Ababa University, Addis Ababa, Ethiopia; 10 PLOS: Public Library of Science, Cambridge, United Kingdom; 11 Initiative for Health Equity Advocacy and Research, Sangath, Bhopal, Madhya Pradesh, India; 12 Cochrane Global Mental Health, University of Verona, Verona, Italy

## Abstract

- Psychosocial rehabilitation is central to recovery-oriented mental health care, yet its uptake remains uneven because of persistent conceptual, methodological, organisational, and implementation barriers.
- Greater conceptual clarity is needed through stakeholder dialogue to establish a common set of core principles that can be adapted across diverse cultural and health system contexts.
- Meaningful co-creation with people with lived experience and their families should be embedded throughout the research and implementation cycle, from priority setting and intervention design to evaluation, scale-up, and dissemination.
- Evidence synthesis should adopt component-based and mechanism-informed approaches to identify the active components of psychosocial rehabilitation interventions and the mechanisms through which they produce change.
- Psychosocial rehabilitation should be recognised as a core component of mental healthcare, integrated from first contact and delivered across the continuum of care.

## Abstract 2

In this Policy Forum article, Eleonora Prima and colleagues outline conceptual, methodological, organisational and implementation barriers to psychosocial rehabilitation, and argue why it should not be confined to secondary or tertiary care settings, but rather recognised as a core component of mental health care.

## Introduction

The global population experiencing limitations in physical, mental, and social functioning is increasing substantially, driven by demographic and epidemiological transitions such as population ageing and the rising prevalence of non-communicable diseases [1]. Although these limitations are not necessarily synonymous with disability, they frequently restrict people’s ability to participate fully in everyday life and therefore create a growing need for rehabilitation [1,2]. According to the World Health Organisation (WHO), rehabilitation comprises interventions designed to optimise functioning and reduce disability by addressing the interaction between individuals and their environments [3]. Accordingly, rehabilitation is increasingly recognised as an essential health strategy rather than a supplementary service [4]. It is now considered a core component of Universal Health Coverage (UHC) and a key contributor to achieving Sustainable Development Goal 3 (SDG3), which aims to ensure healthy lives and well-being for all [5]. The WHO Rehabilitation 2030 call to action has further reinforced its importance by urging countries to strengthen rehabilitation across all levels of care [6].

In mental health, however, rehabilitation presents unique challenges and opportunities. For people living with severe mental health conditions, limitations in functioning arise not only from symptom severity but also from the dynamic interaction between individuals and their social, economic, and political environments [7]. These challenges may be further compounded by intersecting forms of discrimination, stigma, and structural disadvantage [8]. Consequently, promoting mental health and well-being extends beyond symptom reduction; it requires supporting functioning, social inclusion, personal recovery, empowerment, and overall well-being in ways that reflect each individual’s own values, aspirations, and life goals [9]. Rather than focussing solely on restoring functioning or enabling predefined social roles, psychosocial rehabilitation aims to support people in living well in ways they consider meaningful. This includes fostering autonomy, strengthening meaningful relationships and a sense of belonging, and enabling participation in community life in ways that promote both personal fulfilment and social inclusion. Psychosocial rehabilitation has therefore emerged as a key approach to achieving these goals, combining individual, social, and environmental strategies to support recovery-oriented care [10–13].

Over the past decades, a growing body of evidence has demonstrated the effectiveness of psychosocial rehabilitation interventions for people living with severe mental health conditions across diverse settings and resource contexts [13–16]. Interventions can include social skills training to improve interpersonal communication and everyday interactions, cognitive remediation to strengthen cognitive abilities such as memory and attention, individual placement and support to help people find and maintain employment, and community-based rehabilitation and inclusive development to promote participation in everyday community life [14,16–19]. For instance, a meta-analysis of 130 randomised controlled trials (RCTs) involving 8,851 people living with schizophrenia spectrum disorders revealed that cognitive remediation was effective on cognition (effect size (*d*) = 0.29, 95% Confidence Interval (CI) 0.24–0.34) and functioning (*d* = 0.22, 95% CI 0.16–0.29) [20]. Similarly, a meta-analysis of 17 RCTs found that individuals with severe mental health conditions who received individual placement and support were 2.4 times more likely to obtain competitive employment than those who participated in other vocational activities (Relative Risk (RR) = 2.40, 95% CI 1.99–2.90) [19]. At the same time, there has been growing recognition of the harms associated with coercive, institutional, and segregating practices in mental healthcare, reinforcing calls for rights-based and community-oriented approaches [21]. International mental health policies increasingly advocate recovery-oriented, person-centred, and community-based models of care [22,23]. Yet despite this convergence of evidence and human rights principles, psychosocial rehabilitation remains insufficiently prioritised within research, policy, and routine service delivery in many parts of the world. Its implementation is often inconsistent due to differences in how psychosocial rehabilitation is defined, prioritised and integrated into care pathways across mental health systems, as well as continues to receive insufficient attention to cultural and contextual factors, limiting its integration into routine mental healthcare [24]. Additionally, the WHO Mental Health Atlas highlights significant disparities in mental health resources, with median government spending ranging from US$0.04 per capita in low-income countries to US$65.89 in high-income countries. These disparities may affect the availability of trained professionals, community-based services, and other resources needed to implement and sustain psychosocial rehabilitation interventions worldwide [22].

Against this background, we argue that the uneven uptake of psychosocial rehabilitation across countries and health systems is not only due to limited resources or implementation capacity. It also reflects persistent conceptual, methodological, and organisational barriers that continue to shape how psychosocial rehabilitation is understood, studied, and delivered. Although important advances have been made in several countries, including the development of recovery-oriented models, professional standards, and community-based approaches, these developments have not yet been consistently translated across diverse health systems and sociocultural contexts [22,23,25,26]. As a result, psychosocial rehabilitation continues to be conceptualised, studied, and implemented in markedly different ways worldwide.

In this article, we examine four interrelated barriers spanning conceptual, methodological, implementation, and systems-level challenges and propose corresponding action priorities (Table 1). This Policy Forum is intended to provide a starting point for discussion and action and therefore the list of barriers is not exhaustive. Their selection was informed by discussions within a multidisciplinary and international author group, including people with lived experience, researchers, clinicians, and experts in psychosocial rehabilitation across low-, middle-, and high-income settings, and reflects areas considered particularly relevant across diverse contexts.

**Table 1:** Key barriers and proposed solutions for strengthening psychosocial rehabilitation in mental health systems.

Barrier	Proposed solutions
**1. Conceptual and terminological fragmentation** Heterogeneous definitions and terminology, and outdated and decontextualised frameworks, creating uncertainty about the scope, goals, and operational boundaries of psychosocial rehabilitation.	**Consolidate terminology and conceptual principles** Promote dialogue and consensus-building among stakeholders within any given region to develop shared, context-sensitive principles for psychosocial rehabilitation research and practice. *Key actors*: People with lived experience (PWLE) and families, rehabilitation practitioners, clinical researchers, community organisations, policymakers, guideline developers. *Possible indicators*: number of consensus statements developed; presence of agreed core principles in relevant guidelines, standards, and training.
**2. Limited integration of lived experience perspectives** The priorities and perspectives of PWLE and families remain insufficiently reflected in research, outcome selection, and service development.	**Embed meaningful co-creation** Systematically involve PWLE and families in accountable co-production throughout the research and implementation cycle, from priority setting and intervention design to implementation monitoring, evaluation, scale-up, and dissemination. *Key actors*: PWLE and families, researchers, service providers, funders and institutions. *Possible indicators*: number of research projects involving PWLE; number of PWLE employed in mental health services
**3. Limited understanding of active components** The effective components of psychosocial rehabilitation interventions remain poorly understood, limiting evidence-informed decision-making.	**Adopt precision-oriented evidence synthesis** Use component-based and mechanism-informed approaches to identify the most beneficial active components and the mechanisms through which they exert their effects. *Key actors*: researchers, methodologists, clinicians, PWLE and families. *Possible indicators*: open access taxonomy of intervention components; number of publications examining intervention components and mechanisms of action
**4. Delayed integration into care pathways** Psychosocial rehabilitation is often viewed as a specialised, late-stage intervention reserved for individuals with established disability.	**Integrate rehabilitation early in care** Position psychosocial rehabilitation as a core component of mental healthcare, embedded from first contact and delivered across the continuum of care. *Key actors*: policymakers, mental health services, rehabilitation practitioners, community organisations, guideline developers. *Possible indicators*: presence of early psychosocial rehabilitation in relevant guidelines and care pathways; number of services offering psychosocial rehabilitation from first contact.

## Conceptual and terminological fragmentation

Psychosocial rehabilitation has evolved through several influential conceptual frameworks that have informed research, policy, and practice in different countries and service systems [27–31]. As a result, multiple definitions and framings of psychosocial rehabilitation coexist, reflecting heterogeneous and only partially overlapping understandings of the field. The most recent international consensus statement on psychosocial rehabilitation dates back to 1996 [32], while more recent WHO guidance has adopted the broader concept of rehabilitation as “a set of interventions designed to optimise functioning and reduce disability in individuals with health conditions, considering their interaction with the environment” [3]. Although this definition provides a valuable overarching framework, further work is needed to articulate a common set of core principles for psychosocial rehabilitation that can guide research, policy, and practice while remaining sufficiently flexible to be adapted across different cultural, policy, and service contexts.

At its core, psychosocial rehabilitation aims to enhance or protect people’s capacity to live, learn, work, and participate in community life in accordance with the roles, relationships, and aspirations they value, while requiring as little professional support as possible [17,25]. This person-centred perspective positions functioning as a key health outcome and calls for health systems to move beyond purely biomedical models of care [33,34]. However, while functioning provides an important overarching goal for rehabilitation, it does not, by itself, distinguish psychosocial rehabilitation from other interventions that may also improve functioning, including pharmacological, psychological, and broader social interventions. This may also contribute to uncertainty about what falls within the scope of the field, particularly when evidence-based psychosocial interventions pursue rehabilitation-related goals without being explicitly labelled as such. This conceptual uncertainty may partly reflect the historical development of psychosocial rehabilitation during deinstitutionalisation, when it was often associated with helping people develop or regain the skills considered necessary for community living in that historical context. This legacy may still shape perceptions of rehabilitation as being relevant only when skills have been lost, rather than for preventing the loss of skills, which is also within its scope. Greater conceptual clarity is therefore needed to define the distinctive goals, mechanisms, and implementation strategies of psychosocial rehabilitation across diverse mental health systems.

This conceptual diversity is further compounded by uncertainty surrounding the relationship between psychosocial rehabilitation and personal recovery. Personal recovery is widely understood as the individual process through which people pursue a meaningful, hopeful, and self-directed life, regardless of the presence or absence of symptoms [35–38]. From this perspective, personal recovery represents both a process and an overarching goal of psychosocial rehabilitation rather than an intervention in itself. However, the terms rehabilitation and recovery are not used consistently across countries, stakeholder groups, and service traditions. In some settings, psychosocial rehabilitation is embedded within recovery-oriented systems of care, whereas in others, concepts such as recovery, community-based rehabilitation, or inclusive community development are also used to describe similar principles of person-centred, rights-based, and community-oriented support [23]. Rather than reflecting competing paradigms, these differences highlight the need for greater conceptual clarity regarding how these approaches relate to one another while remaining responsive to diverse cultural, policy, and service contexts. This may also warrant consideration of whether current terminology adequately reflects the contemporary scope and recovery-oriented principles of psychosocial rehabilitation [39].

A further implication concerns the scope of psychosocial rehabilitation itself. Rather than being confined to formal treatment settings, psychosocial rehabilitation is increasingly understood as a process that supports people’s participation in the environments where identity, agency, relationships, and meaningful social roles are developed, enabled, and sustained [10,17,36]. These environments extend beyond hospitals and community mental health services to include homes, workplaces, educational settings, and broader community networks. Although psychosocial rehabilitation models have established competencies and professional standards in some settings, their implementation varies considerably across health systems [23,26]. In many contexts, particularly in low- and middle-income countries, psychosocial rehabilitation is delivered through collaborative, community-based models involving mental health professionals, peers, families, community organisations, and, where appropriate, task-sharing with community health workers. However, such approaches require adequate training, supervision, and investment to strengthen existing systems rather than simply transferring additional responsibilities to already overstretched workforces. They often build upon broader initiatives such as community-based rehabilitation and community-based inclusive development [10,18,40,41]. The challenge, therefore, is not whether psychosocial rehabilitation should be delivered beyond clinical services, but how its core principles can be implemented across diverse health systems while remaining responsive to local cultures, resources, and community priorities.

To address this conceptual diversity, the field should work toward a common set of core principles for psychosocial rehabilitation that build on existing psychosocial and psychiatric rehabilitation models while remaining sufficiently flexible to accommodate diverse cultural, policy, and service contexts. These principles should be co-developed with people with lived experience (PWLE) of mental health conditions and other key stakeholders, including families, trained mental health professionals, rehabilitation practitioners, community organisations, policymakers, researchers, and other relevant social actors. Open dialogue and critical debate are essential to achieving greater conceptual clarity and advancing a contemporary vision of psychosocial rehabilitation [42]. In turn, this would strengthen its recognition as a core, cross-cutting component of mental health systems and support its systematic integration across care pathways and throughout the life course. Rather than replacing existing rehabilitation models, this approach seeks to build on their strengths while supporting context-sensitive implementation across diverse health systems.

## Limited integration of lived experience perspectives

Although meaningful involvement of PWLE and families has become an established feature of many psychosocial rehabilitation programmes and research initiatives, it remains inconsistently embedded across countries, disciplines, and service settings [43]. User- and family-led movements have long challenged biomedical models of mental healthcare, advocating approaches that prioritise autonomy, self-determination, meaningful participation, and life goals [44–46]. Peer support is one way in which meaningful involvement of PWLE can be successfully integrated into mental health services and contribute to recovery-oriented care [11,40,47–51]. Recovery colleges are designed to support personal recovery through adult education for people with mental health conditions, emphasising the co-production of service operations and curriculum [12]. However, these principles and approaches are not yet systematically reflected in the service organisation.

Likewise, meaningful involvement in research through participatory action research or co-production remains uneven internationally and rarely extends across the entire research cycle [12,52–54]. As a result, psychosocial rehabilitation research continues to prioritise symptom-related outcomes [55,56], often at the expense of broader outcomes aligned with rights-based and capability-oriented approaches, including empowerment, autonomy, employment, education, and social inclusion, all of which are central to the United Nations Convention on the Rights of Persons with Disabilities [9]. A recent systematic review found that PWLE frequently report that research outcomes fail to capture the issues they consider most important [57]. This disconnect may compromise the relevance, validity, and real-world applicability of research findings, as interventions may be judged effective on outcomes that matter little to those directly affected, while meaningful benefits remain unmeasured.

To address this gap, existing participatory approaches should be strengthened, adapted, and systematically embedded throughout the research and implementation cycle, particularly in settings where meaningful co-production has yet to become routine practice. This requires moving beyond tokenistic consultation toward genuine participatory approaches that enable PWLE and families to shape not only research agendas, but also the development, delivery, and ongoing evaluation of psychosocial rehabilitation interventions [58]. This should be operationalised through the use of explicit co-production methodologies and clear roles and responsibilities for PWLE in decision-making, transparent processes for addressing differing perspectives, and meaningful participation throughout the research cycle. Due attention should be given to accessibility, appropriate recognition of different forms of contribution, and ensuring that family involvement respects the person’s consent, privacy, and safety. Such involvement is essential to ensure that research addresses outcomes that matter to those directly affected and that evidence-based interventions remain relevant, acceptable, and effective in real-world settings.

## Limited understanding of active components

The active ingredients of psychosocial rehabilitation interventions remain poorly defined and understood. Most randomised trials and systematic reviews evaluate individual psychosocial interventions as fixed, indivisible packages. This means that even when interventions comprise multiple components, their effects are usually evaluated at the level of the intervention as a whole. While this approach can determine the overall effectiveness of specific evidence-based interventions, such as social skills training, cognitive remediation, or individual placement and support, it cannot identify the specific contributions of their constituent components, such as training in problem-solving skills, building the therapeutic alliance, or peer-group engagement. Consequently, it remains unclear which elements drive meaningful improvements in functioning, social inclusion, and personal recovery [41,59–61].

To address this challenge, a component-based and mechanism-informed approach to evaluation and evidence synthesis is needed [62]. Such an approach, complemented by qualitative and realist methods that engage relevant stakeholders, would identify the core elements of psychosocial rehabilitation interventions, examine the mechanisms by which they exert their effects, and quantify their contribution to recovery-oriented outcomes [62]. Moving beyond evaluating interventions as fixed packages would provide a more precise understanding of which components are effective, whether individually or in combination, for whom they are most beneficial, and through which mechanisms they produce change. In turn, this would strengthen the evidence base for psychosocial rehabilitation, support more informed intervention selection and referral, and contribute to the development of more efficient, scalable, and context-responsive psychosocial rehabilitation strategies across diverse health systems.

## Delayed integration into care pathways

Although several contemporary community-based models of mental healthcare have successfully integrated psychosocial rehabilitation from the earliest stages of service delivery [63], many mental health systems continue to position rehabilitation as a downstream intervention, introduced only after symptom stabilisation or when disability has become established (2). This sequential model creates a temporal gap that delays access to rehabilitation and reinforces the perception that it is a specialised service reserved for individuals with persistent disability. As a result, psychosocial rehabilitation is often confined to secondary and tertiary care settings and remains peripheral to routine mental health practice [33]. Although contemporary service models increasingly advocate early rehabilitation, this approach has yet to be consistently reflected in clinical guidelines, financing mechanisms, and routine service delivery across many settings [64–66].

To overcome this implementation barrier, successful examples of early, community-based psychosocial rehabilitation should inform broader international efforts to embed rehabilitation from first contact with mental health services, recognising its potential preventive role in the early phases of mental health conditions [67,68], while adapting implementation to local contexts and available resources [69]. Such an approach recognises that symptom severity alone provides only a partial understanding of a person’s functioning, strengths, aspirations, and opportunities for recovery. Early psychosocial rehabilitation can be delivered through community-based models that address the social determinants of mental health, build on individual capabilities, and strengthen access to opportunities that support recovery, including protection from discrimination and participation in education, employment, and community life. When offered early and in ways that reflect individual priorities, psychosocial rehabilitation can support personal recovery, strengthen functioning and participation, and help prevent the accumulation of adverse social and environmental experiences that may contribute to long-term exclusion and disadvantage [44,70].

## Conclusions

Overall, the barriers discussed in this Policy Forum continue to constrain the development, evaluation, implementation, and impact of psychosocial rehabilitation, limiting its ability to realise its full potential within contemporary mental health systems. Although important advances have been made, psychosocial rehabilitation continues to be conceptualised, studied, and implemented in markedly different ways across the world. Building on decades of progress in psychosocial and psychiatric rehabilitation, the next challenge is to strengthen the field’s conceptual foundations by establishing a common set of core principles, including self-determination, human rights, and meaningful participation. These principles can then guide research, policy, and practice while allowing their implementation and delivery to be shaped by diverse cultural, policy, and service contexts. Addressing these barriers is therefore essential to support context-sensitive implementation of psychosocial rehabilitation as a core component of integrated mental healthcare, aligned with global health priorities and universal health coverage goals [3,5,6,71]. Achieving this vision will require coordinated global action, cross-sectoral collaboration, and strong health system stewardship to ensure equitable access to high-quality, recovery-oriented psychosocial rehabilitation for people living with severe mental health conditions.

## Abbreviations

CI: Confidence Interval
PWLE: people with lived experience
RCTs: randomised controlled trials
RR: Relative Risk
SDG3: Sustainable Development Goal 3
UHC: Universal Health Coverage
WHO: World Health Organisation
